# 2-(4-Acetamido­benzene­sulfonamido)­benzoic acid

**DOI:** 10.1107/S1600536811020307

**Published:** 2011-06-04

**Authors:** Shahzad Sharif, Islam Ullah Khan, Tariq Mahmood, Sung Kwon Kang

**Affiliations:** a Materials Chemistry Laboratory, Department of Chemistry, Government College University, Lahore 54000, Pakistan; b Glaxo Smith Kline, 18 KM Feroze Pur Road, Lahore 54000, Pakistan; c Department of Chemistry, Chungnam National University, Daejeon 305-764, Republic of Korea

## Abstract

In the title compound, C_15_H_14_N_2_O_5_S, two similar mol­ecules comprise the asymmetric unit, which are linked by strong inter­molecular C—H⋯π inter­actions. Both mol­ecules are bent, with dihedral angles of 71.94 (16) and 74.62 (15)° between the benzene rings. An intra­molecular N—H⋯O hydrogen bond occurs in each mol­ecule. In the crystal, inter­molecular N—H⋯O and O—H⋯O hydrogen bonds link the mol­ecules into a three-dimensional network.

## Related literature

For our previous studies on sulfonamide derivatives, see: Khan *et al.* (2011 ▶); Sharif *et al.* (2010 ▶). For background to the pharmacological use of sulfonamides, see: Korolkovas (1988 ▶); Mandell & Sande (1992 ▶).
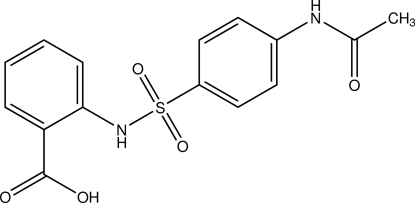


## Experimental

### 

#### Crystal data

C_15_H_14_N_2_O_5_S
*M* *_r_* = 334.34
Monoclinic, 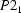

*a* = 9.3721 (19) Å
*b* = 13.036 (3) Å
*c* = 13.132 (3) Å
β = 109.47 (3)°
*V* = 1512.7 (5) Å^3^
*Z* = 4
Mo *K*α radiation
μ = 0.24 mm^−1^
*T* = 296 K
0.25 × 0.12 × 0.09 mm

#### Data collection

Bruker APEXII CCD diffractometer
Absorption correction: multi-scan (*SADABS*; Bruker, 2007 ▶) *T*_min_ = 0.92, *T*_max_ = 0.931
2926 measured reflections
2926 independent reflections
1470 reflections with *I* > 2σ(*I*)

#### Refinement

*R*[*F* ^2^ > 2σ(*F* ^2^)] = 0.045
*wR*(*F* ^2^) = 0.092
*S* = 0.82
2926 reflections
415 parameters
1 restraint
H-atom parameters constrained
Δρ_max_ = 0.26 e Å^−3^
Δρ_min_ = −0.24 e Å^−3^

### 

Data collection: *APEX2* (Bruker, 2007 ▶); cell refinement: *SAINT* (Bruker, 2007 ▶); data reduction: *SAINT*; program(s) used to solve structure: *SHELXS97* (Sheldrick, 2008 ▶); program(s) used to refine structure: *SHELXL97* (Sheldrick, 2008 ▶); molecular graphics: *ORTEP-3 for Windows* (Farrugia, 1997 ▶); software used to prepare material for publication: *WinGX* publication routines (Farrugia, 1999 ▶).

## Supplementary Material

Crystal structure: contains datablock(s) global, I. DOI: 10.1107/S1600536811020307/tk2747sup1.cif

Structure factors: contains datablock(s) I. DOI: 10.1107/S1600536811020307/tk2747Isup2.hkl

Supplementary material file. DOI: 10.1107/S1600536811020307/tk2747Isup3.cml

Additional supplementary materials:  crystallographic information; 3D view; checkCIF report

## Figures and Tables

**Table 1:** Hydrogen-bond geometry (Å, °) *Cg*1 and *Cg*2 are the centroids of the C37–C42 and C14–C19 rings, respectively.

*D*—H⋯*A*	*D*—H	H⋯*A*	*D*⋯*A*	*D*—H⋯*A*
O9—H9⋯O22^i^	0.82	1.84	2.649 (6)	168
N10—H10⋯O8	0.86	2.13	2.624 (7)	116
N20—H20⋯O13^ii^	0.86	2.24	3.073 (6)	164
O31—H31⋯O45^iii^	0.82	1.81	2.623 (6)	174
N33—H33⋯O32	0.86	2.17	2.641 (7)	114
N43—H43⋯O36^i^	0.86	2.11	2.958 (7)	168
C23—H23*B*⋯*Cg*1	0.96	2.74	3.6110 (15)	151
C46—H46*C*⋯*Cg*2	0.96	2.71	3.5821 (13)	151

Symmetry codes: (i) 
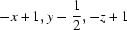
; (ii) 
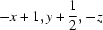
; (iii) 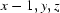.

**Fig. 1.**
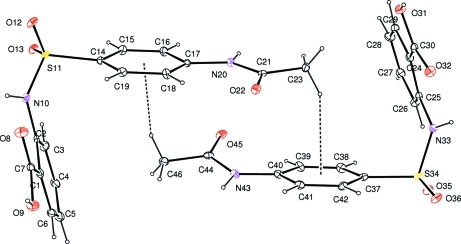
The molecular structures of the two independent molecules of (I) showing the atom-numbering scheme and 30% probability ellipsoids. The C—H···π interactions are shown as dashed lines.

**Fig. 2.**
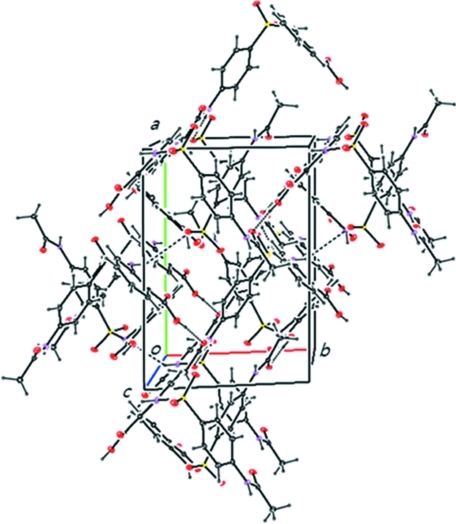
Part of the crystal structure of (I), viewed normal to (0 0 1), illustrating the 3-D network of molecules linked by intermolecular N—H···O and O—H···O hydrogen bonds (dashed lines).

## supplementary crystallographic information

### Comment

In continuation of our structural studies of sulfonamides (Khan *et al.*, 2011; Sharif *et al.*, 2010) of the interest owing to their potential as biologically active molecules (Korolkovas, 1988; Mandell & Sande, 1992), herein, we report the crystal structure of the title compound, (I).

Two independent but similar molecules comprise the asymmetric unit, Fig. 1. The phenyl carboxyl moieties are almost planar with r.m.s. deviations of 0.012 and 0.023 Å from the corresponding least-squares plane defined by the eight constituent atoms. The dihedral angles between the benzene rings are 71.94 (16) and 74.62 (15) °. The two independent molecules are linked by intermolecular C—H···π interactions (centroid—H distance = 2.711 (3) and 2.740 (3) Å) (Fig. 1). In the crystal, intermolecular N—H···O and O—H···O hydrogen bonds link the molecules into a three-dimensional network (Table 1, Fig. 2).

### Experimental

To anthranilic acid (137 mg, 1 mmol) in distilled water (10 ml) was added 4-acetamidobenzenesulfonyl chloride (234 mg, 1 mmol). The pH = 8 was maintained by 3% Na_2_CO_3_ with stirring at room temperature. The reaction was monitored by TLC. After completion of reaction, the solution was adjusted to pH =3 with 3 N HCl solution. The white precipitate that formed was filtered and washed with water. Crystallization was from methanol.

### Refinement

All the H atoms were positioned in their idealized geometries with C—H = 0.93–0.96 Å, N—H = 0.86 Å and O—H = 0.82 Å, and were refined using a riding model with *U*_iso_(H) = 1.2*U*_eq_ for aromatic C and N atoms and with *U*_iso_(H) = 1.5*U*_eq_ for methyl C and O atoms. In the absence of significant anomalous scattering effects, 2267 Friedel pairs have been merged.

### Crystal data

**Table:**

C_15_H_14_N_2_O_5_S	*F*(000) = 696
*M**_r_* = 334.34	*D*_x_ = 1.468 Mg m^−^^3^
Monoclinic, *P*2_1_	Mo *K*α radiation, λ = 0.71073 Å
Hall symbol: P 2yb	Cell parameters from 1295 reflections
*a* = 9.3721 (19) Å	θ = 2.8–18.7°
*b* = 13.036 (3) Å	µ = 0.24 mm^−^^1^
*c* = 13.132 (3) Å	*T* = 296 K
β = 109.47 (3)°	Block, violet
*V* = 1512.7 (5) Å^3^	0.25 × 0.12 × 0.09 mm
*Z* = 4	

### Data collection

**Table:**

Bruker APEXII CCD diffractometer	2926 independent reflections
graphite	1470 reflections with *I* > 2σ(*I*)
φ and ω scans	θ_max_ = 25.5°, θ_min_ = 2.8°
Absorption correction: multi-scan (*SADABS*; Bruker, 2007)	*h* = −11→10
*T*_min_ = 0.92, *T*_max_ = 0.931	*k* = 0→15
2926 measured reflections	*l* = 0→15

### Refinement

**Table:**

Refinement on *F*^2^	Primary atom site location: structure-invariant direct methods
Least-squares matrix: full	Secondary atom site location: difference Fourier map
*R*[*F*^2^ > 2σ(*F*^2^)] = 0.045	Hydrogen site location: inferred from neighbouring sites
*wR*(*F*^2^) = 0.092	H-atom parameters constrained
*S* = 0.82	*w* = 1/[σ^2^(*F*_o_^2^) + (0.0338*P*)^2^] where *P* = (*F*_o_^2^ + 2*F*_c_^2^)/3
2926 reflections	(Δ/σ)_max_ < 0.001
415 parameters	Δρ_max_ = 0.26 e Å^−^^3^
1 restraint	Δρ_min_ = −0.24 e Å^−^^3^

### Special details

**Table:**

Geometry. All s.u.'s (except the s.u. in the dihedral angle between two l.s. planes) are estimated using the full covariance matrix. The cell s.u.'s are taken into account individually in the estimation of s.u.'s in distances, angles and torsion angles; correlations between s.u.'s in cell parameters are only used when they are defined by crystal symmetry. An approximate (isotropic) treatment of cell s.u.'s is used for estimating s.u.'s involving l.s. planes.
Refinement. Refinement of *F*^2^ against ALL reflections. The weighted *R*-factor *wR* and goodness of fit *S* are based on *F*^2^, conventional *R*-factors *R* are based on *F*, with *F* set to zero for negative *F*^2^. The threshold expression of *F*^2^ > 2σ(*F*^2^) is used only for calculating *R*-factors(gt) *etc*. and is not relevant to the choice of reflections for refinement. *R*-factors based on *F*^2^ are statistically about twice as large as those based on *F*, and *R*- factors based on ALL data will be even larger.

### Fractional atomic coordinates and isotropic or equivalent isotropic displacement parameters (Å^2^)

**Table:**

	*x*	*y*	*z*	*U*_iso_*/*U*_eq_	
C1	0.9116 (7)	0.1951 (5)	0.4462 (5)	0.0489 (17)	
C2	0.8856 (6)	0.1876 (4)	0.3361 (5)	0.0378 (15)	
C3	0.9664 (8)	0.2491 (5)	0.2895 (6)	0.069 (2)	
H3	0.9489	0.2433	0.2157	0.082*	
C4	1.0705 (8)	0.3179 (6)	0.3474 (7)	0.068 (2)	
H4	1.124	0.3585	0.3143	0.081*	
C5	1.0948 (8)	0.3260 (6)	0.4565 (7)	0.078 (2)	
H5	1.1645	0.3736	0.4972	0.094*	
C6	1.0181 (8)	0.2652 (6)	0.5064 (6)	0.0547 (18)	
H6	1.0374	0.271	0.5804	0.066*	
C7	0.8274 (7)	0.1297 (5)	0.4990 (5)	0.0444 (16)	
O8	0.7297 (6)	0.0692 (4)	0.4533 (4)	0.0761 (16)	
O9	0.8716 (5)	0.1415 (4)	0.6050 (4)	0.0753 (14)	
H9	0.8211	0.1043	0.63	0.113*	
N10	0.7746 (6)	0.1191 (4)	0.2728 (4)	0.0556 (15)	
H10	0.7657	0.0613	0.3017	0.067*	
S11	0.6629 (2)	0.13965 (13)	0.15141 (13)	0.0496 (5)	
O12	0.5537 (5)	0.0600 (3)	0.1345 (3)	0.0650 (13)	
O13	0.7415 (4)	0.1479 (3)	0.0766 (3)	0.0601 (12)	
C14	0.5771 (6)	0.2586 (5)	0.1545 (5)	0.0409 (16)	
C15	0.5660 (7)	0.3302 (5)	0.0747 (5)	0.0505 (18)	
H15	0.6111	0.3183	0.0225	0.061*	
C16	0.4882 (7)	0.4181 (5)	0.0734 (5)	0.0523 (19)	
H16	0.48	0.4663	0.0196	0.063*	
C17	0.4204 (6)	0.4379 (5)	0.1504 (5)	0.0374 (15)	
C18	0.4315 (7)	0.3678 (5)	0.2282 (5)	0.0485 (18)	
H18	0.3873	0.3809	0.2806	0.058*	
C19	0.5082 (7)	0.2768 (5)	0.2307 (5)	0.0479 (17)	
H19	0.5134	0.228	0.2834	0.057*	
N20	0.3395 (5)	0.5312 (4)	0.1386 (4)	0.0434 (13)	
H20	0.3362	0.5668	0.0827	0.052*	
C21	0.2673 (6)	0.5729 (5)	0.2010 (5)	0.0411 (16)	
O22	0.2672 (5)	0.5316 (3)	0.2864 (3)	0.0580 (13)	
C23	0.1884 (7)	0.6728 (4)	0.1635 (5)	0.059 (2)	
H23A	0.2015	0.6925	0.0967	0.088*	
H23B	0.2307	0.7246	0.217	0.088*	
H23C	0.0825	0.6653	0.1527	0.088*	
C24	−0.0124 (7)	0.9059 (5)	0.1004 (5)	0.0449 (16)	
C25	0.0910 (7)	0.9800 (5)	0.1592 (5)	0.0437 (17)	
C26	0.1722 (7)	1.0380 (5)	0.1075 (6)	0.061 (2)	
H26	0.2361	1.09	0.1449	0.074*	
C27	0.1581 (7)	1.0185 (6)	0.0021 (6)	0.064 (2)	
H27	0.2137	1.0569	−0.0312	0.077*	
C28	0.0617 (8)	0.9422 (6)	−0.0555 (6)	0.071 (2)	
H28	0.0553	0.9272	−0.1261	0.085*	
C29	−0.0240 (7)	0.8892 (5)	−0.0065 (6)	0.0510 (18)	
H29	−0.0924	0.8405	−0.0463	0.061*	
C30	−0.1013 (7)	0.8458 (5)	0.1504 (6)	0.0479 (17)	
O31	−0.1843 (5)	0.7725 (4)	0.0872 (4)	0.0677 (14)	
H31	−0.2313	0.7413	0.12	0.101*	
O32	−0.1066 (5)	0.8583 (4)	0.2407 (4)	0.0691 (15)	
N33	0.1094 (5)	0.9963 (4)	0.2685 (4)	0.0555 (15)	
H33	0.0295	0.9965	0.2869	0.067*	
S34	0.2731 (2)	1.01499 (13)	0.36347 (14)	0.0574 (5)	
O35	0.3439 (5)	1.1036 (3)	0.3377 (4)	0.0690 (14)	
O36	0.2365 (5)	1.0137 (4)	0.4614 (3)	0.0713 (13)	
C37	0.3874 (7)	0.9084 (5)	0.3622 (5)	0.0471 (17)	
C38	0.4644 (7)	0.9058 (5)	0.2911 (6)	0.059 (2)	
H38	0.4576	0.9616	0.2457	0.07*	
C39	0.5520 (7)	0.8235 (6)	0.2842 (6)	0.0584 (19)	
H39	0.6053	0.8234	0.2358	0.07*	
C40	0.5584 (7)	0.7408 (5)	0.3516 (5)	0.0498 (18)	
C41	0.4833 (7)	0.7435 (5)	0.4263 (5)	0.0540 (19)	
H41	0.4912	0.6889	0.4733	0.065*	
C42	0.3979 (7)	0.8271 (5)	0.4301 (5)	0.0530 (18)	
H42	0.3462	0.8289	0.4794	0.064*	
N43	0.6463 (5)	0.6529 (4)	0.3513 (4)	0.0563 (15)	
H43	0.6701	0.6161	0.4089	0.068*	
C44	0.6993 (7)	0.6179 (6)	0.2710 (6)	0.0548 (19)	
O45	0.6655 (5)	0.6604 (4)	0.1829 (4)	0.0682 (14)	
C46	0.7926 (7)	0.5216 (6)	0.2971 (5)	0.071 (2)	
H46A	0.8045	0.5004	0.3695	0.106*	
H46B	0.8903	0.5345	0.291	0.106*	
H46C	0.7426	0.4684	0.2475	0.106*	

### Atomic displacement parameters (Å^2^)

**Table:**

	*U*^11^	*U*^22^	*U*^33^	*U*^12^	*U*^13^	*U*^23^
C1	0.055 (4)	0.049 (5)	0.045 (4)	0.016 (4)	0.019 (4)	0.011 (4)
C2	0.038 (4)	0.027 (4)	0.051 (4)	0.014 (3)	0.019 (4)	0.012 (4)
C3	0.077 (6)	0.060 (5)	0.071 (6)	−0.005 (4)	0.027 (5)	0.003 (4)
C4	0.050 (5)	0.063 (6)	0.081 (6)	−0.001 (4)	0.009 (5)	0.017 (5)
C5	0.063 (6)	0.059 (6)	0.094 (7)	−0.003 (4)	0.002 (5)	0.006 (5)
C6	0.049 (4)	0.051 (5)	0.057 (5)	−0.004 (4)	0.007 (4)	0.004 (4)
C7	0.050 (4)	0.048 (4)	0.035 (4)	0.010 (4)	0.013 (3)	0.004 (4)
O8	0.091 (4)	0.081 (4)	0.056 (3)	−0.030 (3)	0.023 (3)	0.005 (3)
O9	0.083 (4)	0.088 (4)	0.054 (3)	−0.007 (3)	0.022 (3)	0.003 (3)
N10	0.081 (4)	0.034 (3)	0.046 (3)	0.006 (3)	0.014 (3)	0.008 (3)
S11	0.0681 (12)	0.0425 (11)	0.0387 (10)	−0.0015 (10)	0.0186 (9)	−0.0069 (9)
O12	0.101 (4)	0.038 (3)	0.063 (3)	−0.023 (3)	0.038 (3)	−0.013 (2)
O13	0.079 (3)	0.056 (3)	0.057 (3)	−0.001 (3)	0.038 (3)	−0.010 (3)
C14	0.044 (4)	0.036 (4)	0.041 (4)	0.002 (3)	0.011 (3)	0.001 (3)
C15	0.073 (5)	0.053 (5)	0.038 (4)	0.003 (4)	0.035 (4)	0.007 (4)
C16	0.065 (5)	0.054 (5)	0.040 (4)	0.011 (4)	0.020 (4)	0.015 (3)
C17	0.041 (4)	0.033 (4)	0.039 (4)	0.001 (3)	0.014 (3)	0.002 (3)
C18	0.055 (4)	0.052 (5)	0.051 (4)	0.010 (3)	0.033 (4)	0.018 (4)
C19	0.064 (4)	0.052 (5)	0.033 (4)	−0.004 (4)	0.023 (4)	0.013 (4)
N20	0.051 (3)	0.048 (4)	0.037 (3)	0.004 (3)	0.023 (3)	0.012 (3)
C21	0.043 (4)	0.042 (4)	0.034 (4)	−0.011 (3)	0.006 (3)	−0.005 (3)
O22	0.077 (3)	0.058 (3)	0.051 (3)	0.004 (3)	0.036 (3)	0.001 (3)
C23	0.069 (5)	0.052 (5)	0.052 (4)	0.014 (4)	0.017 (4)	−0.008 (4)
C24	0.043 (4)	0.048 (4)	0.045 (4)	0.004 (3)	0.016 (3)	0.011 (4)
C25	0.046 (4)	0.048 (5)	0.042 (4)	0.013 (3)	0.020 (4)	0.009 (3)
C26	0.065 (5)	0.061 (5)	0.058 (5)	−0.001 (4)	0.022 (4)	−0.004 (4)
C27	0.065 (5)	0.064 (6)	0.063 (5)	0.008 (4)	0.023 (4)	0.026 (5)
C28	0.068 (5)	0.089 (7)	0.053 (5)	−0.002 (5)	0.017 (4)	0.008 (5)
C29	0.055 (5)	0.056 (5)	0.043 (4)	0.007 (4)	0.017 (4)	0.004 (4)
C30	0.052 (5)	0.046 (5)	0.048 (5)	0.013 (4)	0.021 (4)	0.010 (4)
O31	0.071 (3)	0.070 (3)	0.064 (3)	−0.015 (3)	0.025 (3)	−0.010 (3)
O32	0.080 (4)	0.076 (4)	0.065 (3)	−0.010 (3)	0.041 (3)	0.000 (3)
N33	0.051 (3)	0.070 (4)	0.046 (3)	0.009 (3)	0.016 (3)	−0.011 (3)
S34	0.0712 (13)	0.0507 (13)	0.0499 (11)	−0.0118 (10)	0.0194 (10)	−0.0137 (10)
O35	0.088 (4)	0.042 (3)	0.079 (3)	−0.022 (3)	0.029 (3)	−0.008 (3)
O36	0.101 (4)	0.064 (3)	0.056 (3)	−0.014 (3)	0.037 (3)	−0.017 (3)
C37	0.055 (5)	0.047 (5)	0.038 (4)	−0.011 (3)	0.013 (4)	−0.002 (4)
C38	0.064 (5)	0.052 (5)	0.062 (5)	−0.010 (4)	0.024 (4)	0.024 (4)
C39	0.052 (5)	0.067 (5)	0.062 (5)	0.007 (4)	0.027 (4)	0.026 (4)
C40	0.041 (4)	0.065 (5)	0.045 (4)	−0.001 (4)	0.015 (4)	0.008 (4)
C41	0.058 (5)	0.065 (6)	0.038 (4)	−0.002 (4)	0.014 (4)	0.010 (4)
C42	0.063 (5)	0.059 (5)	0.042 (4)	−0.004 (4)	0.025 (4)	−0.012 (4)
N43	0.058 (4)	0.062 (4)	0.045 (3)	0.003 (3)	0.012 (3)	0.019 (3)
C44	0.047 (4)	0.066 (6)	0.048 (5)	−0.021 (4)	0.013 (4)	−0.005 (4)
O45	0.098 (4)	0.065 (4)	0.050 (3)	−0.020 (3)	0.036 (3)	−0.006 (3)
C46	0.057 (5)	0.084 (6)	0.066 (5)	0.004 (5)	0.014 (4)	−0.015 (5)

### Geometric parameters (Å, °)

**Table:**

C1—C2	1.387 (8)	C24—C29	1.389 (8)
C1—C6	1.389 (9)	C24—C25	1.403 (8)
C1—C7	1.482 (8)	C24—C30	1.450 (8)
C2—C3	1.379 (8)	C25—C26	1.399 (8)
C2—N10	1.412 (7)	C25—N33	1.402 (7)
C3—C4	1.356 (9)	C26—C27	1.370 (8)
C3—H3	0.93	C26—H26	0.93
C4—C5	1.378 (9)	C27—C28	1.386 (9)
C4—H4	0.93	C27—H27	0.93
C5—C6	1.374 (9)	C28—C29	1.372 (9)
C5—H5	0.93	C28—H28	0.93
C6—H6	0.93	C29—H29	0.93
C7—O8	1.206 (7)	C30—O32	1.215 (7)
C7—O9	1.322 (7)	C30—O31	1.332 (8)
O9—H9	0.82	O31—H31	0.82
N10—S11	1.611 (5)	N33—S34	1.641 (5)
N10—H10	0.86	N33—H33	0.86
S11—O13	1.415 (4)	S34—O35	1.429 (4)
S11—O12	1.423 (4)	S34—O36	1.438 (4)
S11—C14	1.754 (6)	S34—C37	1.758 (7)
C14—C19	1.380 (7)	C37—C38	1.358 (8)
C14—C15	1.382 (8)	C37—C42	1.366 (8)
C15—C16	1.355 (8)	C38—C39	1.371 (9)
C15—H15	0.93	C38—H38	0.93
C16—C17	1.386 (7)	C39—C40	1.383 (8)
C16—H16	0.93	C39—H39	0.93
C17—C18	1.348 (8)	C40—C41	1.385 (8)
C17—N20	1.414 (7)	C40—N43	1.412 (7)
C18—C19	1.382 (8)	C41—C42	1.363 (8)
C18—H18	0.93	C41—H41	0.93
C19—H19	0.93	C42—H42	0.93
N20—C21	1.340 (7)	N43—C44	1.385 (8)
N20—H20	0.86	N43—H43	0.86
C21—O22	1.244 (6)	C44—O45	1.224 (7)
C21—C23	1.497 (8)	C44—C46	1.503 (9)
C23—H23A	0.96	C46—H46A	0.96
C23—H23B	0.96	C46—H46B	0.96
C23—H23C	0.96	C46—H46C	0.96
			
C2—C1—C6	118.9 (7)	C29—C24—C25	118.1 (6)
C2—C1—C7	120.8 (6)	C29—C24—C30	120.7 (7)
C6—C1—C7	120.3 (7)	C25—C24—C30	121.2 (6)
C3—C2—C1	119.3 (6)	C26—C25—N33	120.9 (6)
C3—C2—N10	120.8 (6)	C26—C25—C24	119.5 (6)
C1—C2—N10	119.8 (6)	N33—C25—C24	119.6 (6)
C4—C3—C2	122.3 (7)	C27—C26—C25	120.4 (6)
C4—C3—H3	118.9	C27—C26—H26	119.8
C2—C3—H3	118.9	C25—C26—H26	119.8
C3—C4—C5	118.2 (8)	C26—C27—C28	120.7 (7)
C3—C4—H4	120.9	C26—C27—H27	119.7
C5—C4—H4	120.9	C28—C27—H27	119.7
C6—C5—C4	121.3 (7)	C29—C28—C27	118.8 (7)
C6—C5—H5	119.3	C29—C28—H28	120.6
C4—C5—H5	119.3	C27—C28—H28	120.6
C5—C6—C1	119.9 (7)	C28—C29—C24	122.4 (7)
C5—C6—H6	120.1	C28—C29—H29	118.8
C1—C6—H6	120.1	C24—C29—H29	118.8
O8—C7—O9	121.3 (6)	O32—C30—O31	120.0 (6)
O8—C7—C1	125.3 (6)	O32—C30—C24	125.8 (7)
O9—C7—C1	113.4 (6)	O31—C30—C24	114.2 (6)
C7—O9—H9	109.5	C30—O31—H31	109.5
C2—N10—S11	125.8 (4)	C25—N33—S34	124.4 (4)
C2—N10—H10	117.1	C25—N33—H33	117.8
S11—N10—H10	117.1	S34—N33—H33	117.8
O13—S11—O12	117.5 (3)	O35—S34—O36	119.3 (3)
O13—S11—N10	112.5 (3)	O35—S34—N33	109.3 (3)
O12—S11—N10	103.2 (3)	O36—S34—N33	103.7 (3)
O13—S11—C14	107.3 (3)	O35—S34—C37	107.7 (3)
O12—S11—C14	109.7 (3)	O36—S34—C37	109.1 (3)
N10—S11—C14	106.0 (3)	N33—S34—C37	107.1 (3)
C19—C14—C15	119.9 (6)	C38—C37—C42	119.3 (6)
C19—C14—S11	119.9 (5)	C38—C37—S34	119.4 (6)
C15—C14—S11	119.9 (5)	C42—C37—S34	121.2 (5)
C16—C15—C14	119.0 (6)	C37—C38—C39	122.1 (6)
C16—C15—H15	120.5	C37—C38—H38	118.9
C14—C15—H15	120.5	C39—C38—H38	118.9
C15—C16—C17	121.7 (6)	C38—C39—C40	117.8 (6)
C15—C16—H16	119.2	C38—C39—H39	121.1
C17—C16—H16	119.2	C40—C39—H39	121.1
C18—C17—C16	119.2 (6)	C39—C40—C41	120.6 (7)
C18—C17—N20	124.7 (5)	C39—C40—N43	122.2 (6)
C16—C17—N20	116.1 (5)	C41—C40—N43	117.2 (6)
C17—C18—C19	120.5 (6)	C42—C41—C40	119.3 (6)
C17—C18—H18	119.7	C42—C41—H41	120.3
C19—C18—H18	119.7	C40—C41—H41	120.3
C14—C19—C18	119.7 (6)	C41—C42—C37	120.8 (6)
C14—C19—H19	120.1	C41—C42—H42	119.6
C18—C19—H19	120.1	C37—C42—H42	119.6
C21—N20—C17	130.0 (5)	C44—N43—C40	128.8 (6)
C21—N20—H20	115	C44—N43—H43	115.6
C17—N20—H20	115	C40—N43—H43	115.6
O22—C21—N20	121.9 (6)	O45—C44—N43	121.7 (7)
O22—C21—C23	121.6 (6)	O45—C44—C46	122.7 (7)
N20—C21—C23	116.5 (6)	N43—C44—C46	115.5 (6)
C21—C23—H23A	109.5	C44—C46—H46A	109.5
C21—C23—H23B	109.5	C44—C46—H46B	109.5
H23A—C23—H23B	109.5	H46A—C46—H46B	109.5
C21—C23—H23C	109.5	C44—C46—H46C	109.5
H23A—C23—H23C	109.5	H46A—C46—H46C	109.5
H23B—C23—H23C	109.5	H46B—C46—H46C	109.5

### Hydrogen-bond geometry (Å, °)

**Table:**

*D*—H···*A*	*D*—H	H···*A*	*D*···*A*	*D*—H···*A*
O9—H9···O22^i^	0.82	1.84	2.649 (6)	168
N10—H10···O8	0.86	2.13	2.624 (7)	116
N20—H20···O13^ii^	0.86	2.24	3.073 (6)	164
O31—H31···O45^iii^	0.82	1.81	2.623 (6)	174
N33—H33···O32	0.86	2.17	2.641 (7)	114
N43—H43···O36^i^	0.86	2.11	2.958 (7)	168
C23—H23B···Cg1	0.96	2.74	3.6110 (15)	151
C46—H46C···Cg2	0.96	2.71	3.5821 (13)	151

Symmetry codes: (i) −*x*+1, *y*−1/2, −*z*+1; (ii) −*x*+1, *y*+1/2, −*z*; (iii) *x*−1, *y*, *z*.
